# Investigating and Evaluating Novel Fly Ash-Based Proppant Compressive Strength Under Various Environmental Conditions

**DOI:** 10.3390/ma18020399

**Published:** 2025-01-16

**Authors:** Raz Haydar, Sherif Fakher

**Affiliations:** Department of Petroleum and Energy Engineering, The American University in Cairo, New Cairo 11835, Egypt

**Keywords:** proppant, fly ash, hydraulic fracturing, reservoir stimulation, byproduct, alkaline binder, compressive strength, environmental conditions, resilience, durability

## Abstract

As hydraulic fracturing becomes increasingly prevalent in the oil and gas industry, there is a growing need to develop more cost-effective and sustainable technologies, particularly concerning the materials used. Proppants play a vital role in hydraulic fracturing by ensuring that fractures remain conductive and can withstand the pressure exerted by the surrounding strata. One key parameter for evaluating proppants is their compressive strength, especially under harsh environmental conditions. High-strength proppants, such as those made from ceramics or bauxite, are typically expensive due to the materials and complex manufacturing processes involved. In contrast, fly ash, a byproduct of coal-fired power plants, offers a more affordable and environmentally sustainable alternative for proppant production. This study focuses on the development and evaluation of a fly ash-based proppant, exposed to harsh conditions including high temperature and pressure, as well as acidic, alkaline, saline, and crude oil environments. The fly ash was activated using an alkaline solution, which served as a chemical binder for the proppant. After exposure to these conditions, the compressive strength of the fly ash-based proppants was compared to control samples. The results showed that the proppants’ compressive strength was largely unaffected by the harsh environments, particularly for the B20W25 mix design. However, while the fly ash-based proppants performed well under stress, their compressive strength was still lower than that of conventional proppants used in the industry. The B20W25 sample demonstrated a compressive strength of 1181.19 psi (8.1 MPa), which, although resilient, remains below industry standards.

## 1. Introduction

Proppants are essential components in hydraulic fracturing operations, designed to withstand the closure and overburden pressures generated by subsurface strata [1]. These materials are injected into fractures to maintain long-term conductivity, ensuring the fractures remain open and capable of stimulating reservoirs, particularly those with low permeability, such as unconventional reservoirs and shale formations [2]. The shape, size, and packing pattern of the proppants are key characteristics that influence their performance. The material composition of the proppant significantly impacts its chemical, physical, and mechanical properties, including buoyancy, compressive strength, resilience, and durability [2]. Proppants can be classified as natural, such as sand, or as manufactured products, like ceramics [3]. The degradation of proppant performance may occur due to factors such as diagenesis, embedment, and crushing, which are influenced by the type of proppant and the material it is made from [4,5,6,7]. The primary criterion for evaluating proppants is their compressive strength, which allows them to be categorized based on their ability to resist fracture closure. Natural sand is the most commonly used proppant in the industry, largely due to its low cost and widespread availability [8]. However, depending on the quality of the sand, its crushing strength typically ranges up to 40 MPa, or approximately 5800 psi [9]. As a natural proppant, sand does not undergo significant treatment, and its non-uniform size, shape, and angularity make it highly susceptible to breakage and erosion, which can lead to turbidity. Moreover, because sand proppants are unconsolidated sandstones, they are prone to deterioration when exposed to hydrochloric acid (HCl) injection, reducing their strength and effectiveness [8,10].

Resin-coated sand proppants offer a significant improvement over traditional sand by withstanding higher compressive stresses, typically exceeding 6000 psi, while also eliminating turbidity and chemical reactivity associated with untreated sand [11,12]. The resin coating enhances the roundness and sphericity of the sand particles, further improving their performance [12]. Sintered proppants, which are typically made from bauxite or kaolin clay, are produced through a high-temperature sintering process that fuses the particles together to eliminate porosity, resulting in a more durable and dense proppant [8]. As a consequence of this manufacturing process and the raw materials used, ceramic and bauxite-based proppants exhibit high density and compressive strength [13,14]. Ceramic proppants are available in various densities: lightweight ceramic proppants can withstand pressures up to 7000 psi, intermediate-density ceramics can endure up to 10,000 psi, and high-density ceramics can resist compressive forces as high as 100 MPa (15,000 psi) [15,16]. Some ceramic proppants are further treated with fluorinated coatings, such as those applied to porous silicone-based ceramics with specific gravities ranging from 1 to 2.2 g/cm³, and strengths between 2000 and 12,000 psi [15]. Ultra-lightweight (ULW) proppants, which include sand, ceramic, and nutshell varieties, are often treated and coated with various polymers, such as resins, epoxies, and multicomponent polymers [1]. Commercially available ULW resin-coated ceramic proppants, such as ULW-1.75 and ULW-1.25, as well as chemically treated walnut shell proppants, have been shown to resist fracture stresses up to 8000 psi at high temperatures [1,17]. Fly ash, a byproduct of coal combustion, is a pozzolanic material composed of spherically shaped aluminum silicate particles [18,19]. As an environmental pollutant, particularly for air and water quality, due to its particle fineness which ranges from 5 to 200 µm depending on the source, fly ash and coal bottom ash have been utilized for decades in the construction industry as an admixture in concrete to improve its properties and as a soil stabilization agent [20,21,22,23,24]. Utilizing fly ash and coal bottom ash to partially substitute cement percentage utilized in mortar and concrete production to mitigate climate change has been researched by many including Menéndez and his colleagues [22]. It should be noted that fly ash is a naturally occurring radioactive material (NORM material), and the finer the particles, the higher the radioactivity is, especially if they are smaller than 45 µm [23]. However, as per a study conducted in a span of 10 years for 765 samples of fly ash by Sanjuán and his team, fly ash is a safe material to be used for manufacturing any building materials in all scenarios considered, as throughout all sampling, the annual effective dose was lower than 1 mSv y^−1^ [23]. In proppant development, fly ash has also been explored as a filler material to enhance proppant properties, along with other materials such as potassium feldspar and manganese oxide [1]. High-alumina fly ash has been incorporated into ceramic proppant manufacturing, in combination with clay and bauxite, via high-temperature sintering processes [25,26]. Proppants produced from fly ash and zinc oxide have demonstrated compressive strengths of approximately 28 MPa (4000 psi) [27].

This research explores fly ash, a byproduct of combustion, as a sustainable and cost-effective alternative for proppants in hydraulic fracturing. Previous studies suggest that fly ash can meet the high-performance demands of hydraulic fracturing in density, environmental resistance and durability, and high-temperature and pressure conditions, making it a promising material for further development [28]. The researchers have indicated that there is a comparable strength between traditional ceramic proppants and ceramic proppants that are composites from fly ash, alum, and bauxite sintered at 1300–1500 °C [29]. Fly ash was also used as an additive component along with low-grade bauxite in a high-silicon proppant to weaken the crystal transformation of silicon dioxide, SiO_2_, during the cooling process when manufacturing the proppant [29]. The proppant’s bulk density was 1.352 g/cm³, with an acid solubility of 4.2% and a 5.3% breaking ratio under a 35 MPa load [29]. Additionally, adopting fly ash in proppant manufacturing aligns with the global shift towards sustainability [30,31]. This shift will allow the petroleum industry to minimize waste and adopt more environmentally responsible practices for greener industrial development [30,31]. Thus, the integration of fly ash into proppant production presents a promising solution for the petroleum industry [31,32]. It not only addresses cost and environmental concerns, but also offers comparable performance to traditional proppants [32,33]. As unconventional reservoirs now dominate global production, with hydraulic fracturing being essential to their stimulation, the need for durable and effective proppants is growing [34]. While sand is the most cost-effective proppant, it struggles in harsh environments, prompting the use of more durable alternatives like ceramics and sintered bauxite. Fly ash offers significant environmental benefits by reducing disposal issues and can be used to create proppants that are corrosion- and erosion-resistant [28]. As of now, fly ash has not been investigated as a standalone material that has been activated chemically through an alkaline solution. All previous studies have used fly ash as a filler material or an additive/admixture to enhance the proppant. Its properties, similar to cement, make it a viable option for manufacturing eco-friendly proppants with comparable performance to traditional materials [18,20]. Moreover, fly ash reduces production costs, aligning with the petroleum industry’s shift towards sustainability. The goal of this research is to develop fly ash-based proppants with high compressive strength before and after chemical and environmental assessments in order to make them ideal for enhancing hydraulic fracturing processes.

## 2. Experimental Description

### 2.1. Experiment Materials

The samples to conduct this experiment were formed by the utilization of the materials below:

- Type F fly ash (FA), a commercially obtained gray powder that chemically consists mainly of silicon oxide (SiO_2_), alumina oxide (Al_2_O_3_), and iron oxide (Fe_2_O_3_) with low calcium oxide (CaO), from Sika (Baar, Switzerland).
- Tap water from New Cairo, Cairo, Egypt.
- Sodium metasilicate (Na_2_SiO_3_), used as an alkaline binder, which is an industrial white granule material, commercially obtained, from Sigma-Aldrich (St. Louis, MO, USA).

The materials that were used for the environmental exposures were the following:

- Hydrochloric acid (HCl), from Sisco Research Laboratories (Mumbai, India).
- Sodium hydroxide (NaOH), from Sisco Research Laboratories (Mumbai, India).
- Sodium chloride (NaCl),from Sisco Research Laboratories (Mumbai, India).
- Crude oil: A highly viscous liquid form of a mixture of hydrocarbons.
- Distilled water (DI Water).
- Heated water bath.
- Carbon dioxide gas (CO_2_).

### 2.2. Experiment Methodology (Formulation and Procedure)

#### 2.2.1. Mix Design

To obtain the samples, fly ash was mixed with an alkaline solution to activate the FA. Na_2_SiO_3_ mixed with tap water provides a strong alkaline binder solution for the FA. The preparation method was conducted based on previous studies published in the literature [18,33,34,35]. The trials of finding a suitable mix design started with firstly mixing different ratios of water and binder, varying from a 15–35% water weight ratio to a 5–30% binder ratio of the total slurry weight percent, meaning that the binder solution Na_2_SiO_3_ content varied from 12 to 50%. After many trials with various binder solution ratios and FA to form a geopolymer slurry mixture, three mix designs were formulated to be tested. The binder solution and geopolymer slurry formulas were as follows:

Binder Solution = Water Weight Ratio (%) + Na_2_SiO_3_ Weight Ratio (%)

Geopolymer Slurry = Fly Ash Weight Ratio (%) + Binder Solution Weight Ratio (%)

The mix designs determined the binder solution and geopolymer slurry weight ratios, where the water, sodium metasilicate, and fly ash were weighted accordingly. The main aim with the initial mix designs was for the slurry to be pourable and cure/harden within one day in ambient conditions. The second aim was for the samples not to crack or be easily broken. The trials resulted into three mix designs that yielded the best samples. To notate the mix designs, the total geopolymer slurry weight ratios were put as indicators and the letters W, standing for water, and B, standing for binder, were used. The water weight percentage was fixed between all samples at 25% of the total weight ratio, and the binder varied between 20%, 22%, and 25% weight percent. Therefore, the mix designs were the following:

- B20W25: binder 20% weight ratio and water 25% weight ratio.
- B22W25: binder 22% weight ratio and water 25% weight ratio.
- B25W25: binder 25% weight ratio and water 25% weight ratio.

#### 2.2.2. Mixing, Sample Formation, and Curing Process

**Figure 1:**
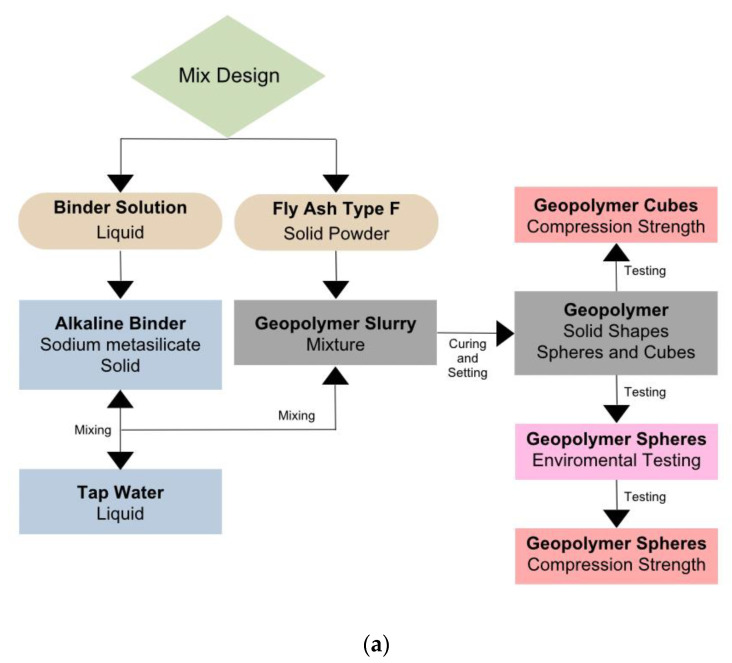

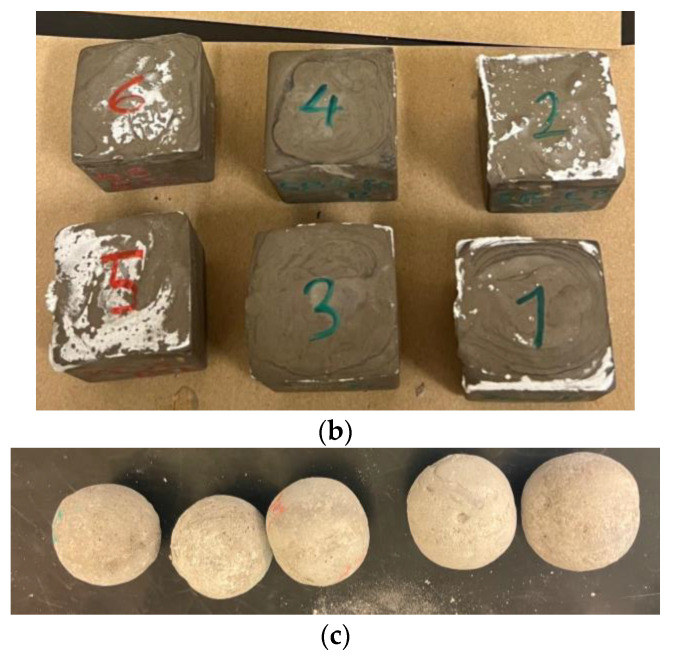
(**a**) Mixing procedure of forming the geopolymer slurry and testing steps. (**b**) Cubical samples. (**c**) Spherical samples.

As illustrated in Figure 1a, the procedure of mixing the geopolymer slurry was broken down into two steps, which are forming the binder solution composed of the alkaline binder and tap water, then incorporating the fly ash into the mixture to form the slurry. The volume of the sample molds were estimated to define the weight of the mix design components. The binder solution, i.e., the water and Na_2_SiO_3_, was weighted according to the mix design and then placed in the mixer to be blended for 1 min or until most of the binder was dissolved. Then, fly ash was weighted and batch-by-batch added to the solution to create a smooth mixture. Between the batches, the mixture was manually mixed to ensure that all the fly ash was incorporated. For 2 to 3 min, the slurry was mixed until a smooth and pourable mixture was ensured. The slurry was poured into cubical and spherical samples. The cubical molds were 5 × 5 × 5 cm, and the spherical molds had a 4.5 cm diameter. The spherical molds were two-part silicone molds where the slurry was poured to one part of the mold and then closed by the other half. The second half of the mold, i.e., the top side, had a small opening where the slurry was later slowly poured in to fill the mold completely. The molds were moved around and shaken manually to ensure that they were filled properly. When the silicone molds were filled properly, the samples were spherical and round with the mold’s standard diameter, which is 4.5 cm. After demolding, if there was any access around the mold closure seams, the excess material was removed gently using a blade. The main reason for the choice of cubical sample dimensions was to be compatible with the testing procedure, standards, and machinery available in our lab. Also, proppants are very small spheres, and thus larger spherical samples were also synthesized to observe their mechanical bearing and their failure shape and behavior. The samples were added to the molds, allowed to harden for 24 h, and demolded. The cubical samples are shown in Figure 1b and the spherical ones in Figure 1c. Afterwards, the spherical samples were put in different media to subject them to various environmental conditions. The samples were kept in the environmental conditions for 5 consecutive days and then retrieved to test their compression strength. The environmental conditions the samples were subjected to were the following: air (ambient conditions being an atmospheric pressure of 14.7 psi, a temperature of 20 °C, and humidity of 30%) and distilled water (controlled environments); CO_2_ gas pressure chamber 500 psi and 900 psi (pressure conditions where a high-pressure filter press machine was used to control and maintain the pressure consistently); 60 °C and 95 °C water bath (elevated-temperature conditions where a electronic thermometer was controlling the consistency of the temperature inside the water bath); HCl: 5% and 15% concentration solution (acidic environments); NaOH: 5% and 15% concentration solution (alkaline environments); NaCl: 10% and 20% concentration solution (saline environments); and crude oil (viscous oil environment). If the samples were placed in a solution or even water for the DI water or temperature testing, they were fully submerged. If the samples were not under elevated temperature and pressure testing conditions, the temperature and pressure were ambient conditions.

### 2.3. Experimental Scope

**Figure 2:**
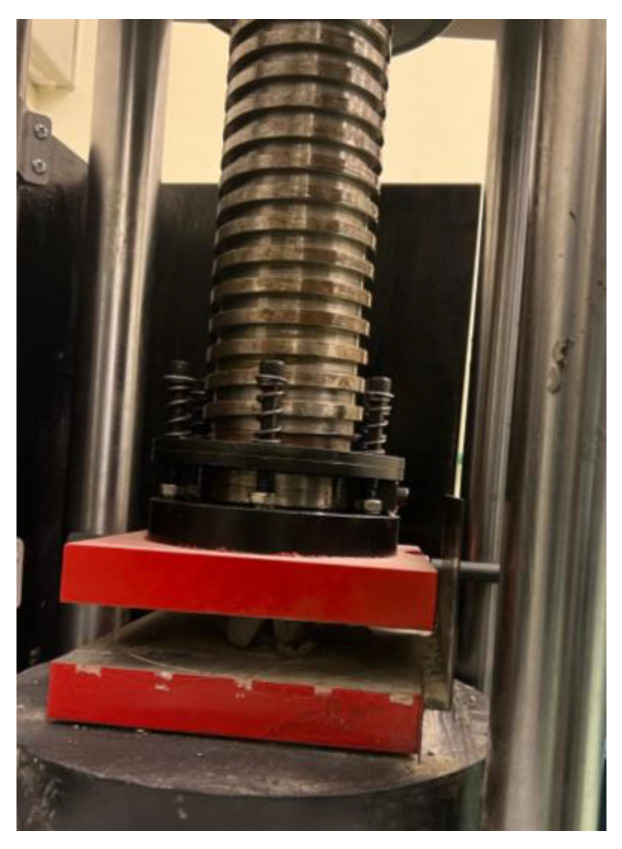
Compression test machine.

The next step after the environmental exposure testing was to test the samples for their load bearing and compression strength, as shown in Figure 1a. The samples’ strength was tested according to and adhering to ASTM C109 for the compression strength of cementitious materials [36]. The test was conducted by putting the samples under a compression machine, seen in Figure 2, where they were put under compression force from two plates stimulating how they will be compressed in the fracture. The universal testing machine used to test the samples was initially calibrated through two steps. First, the device was calibrated based on the size of the samples and their dimensions. Secondly, since the device had no set-in function for geopolymer cement, a new function was added that could be used to measure the compressive strength of geopolymer cement. This was done manually through the testing of multiple samples and through data obtained from the literature. The results were consistent with those found in the literature after testing, and therefore this function was used across all the experiments. The loading rate speed was 0.3 KN/s, and the plate material was stainless steel, as shown in Figure 2. The cubical samples were made to assess a direct compression strength evaluation. Each mix design had five samples and they were cured at room temperature and pressure. Also, two spherical samples of each mix design were compressed after being exposed to different environmental conditions. Every data point presented was an algorithmic average of three samples tested; however, in order to obtain a concise data representation, the average value was plotted.

## 3. Results

### 3.1. Cubical Samples

**Figure 3:**
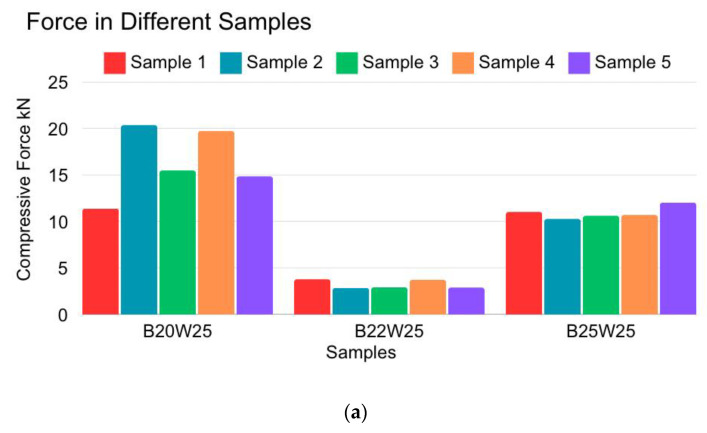

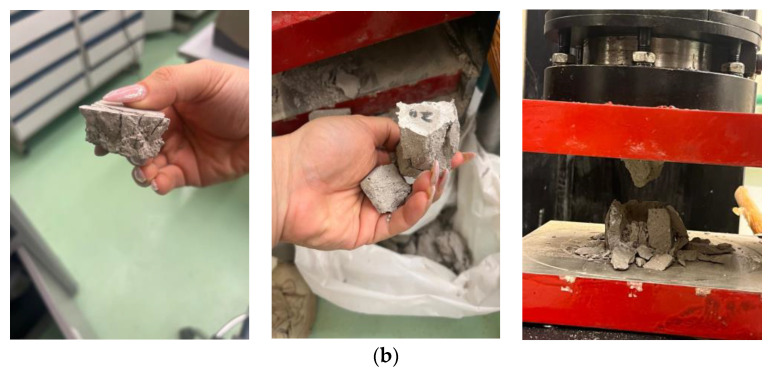
(**a**) Cubical sample controlled environment (air curing) compression force for different samples. (**b**) Final condition of the cubical samples after compression test failure.

By viewing the overall results of the cubical samples, shown in Figure 3a, it is evident that B20W25 withstands the highest compressive force. The compressive strength of the highest performing cubical sample is sample 2 (teal colored bar) of the B20W25 cubes, which are 5 × 5 cm cross-section samples. B20W25 sample 2 withstood 20.36 KN, as indicated in Figure 3a, which is calculated to be 8144 KPa (KN/m²) or 1181.19 psi compression strength. The B20W25 samples exhibited overall higher strength than the other two mix designs. The B22W25 samples had the lowest strength, followed by the B25W25 samples, namely, between 150 and 700 psi. The B22W25 samples crumbled into chunks, as shown in the third picture in Figure 3b, while the B20W25 samples were compressed before failing, as shown in the first two pictures in Figure 3b. This indicates that the binding strength in the B20W25 samples was higher than that in all other samples.

### 3.2. Spherical Samples

**Figure 4:**
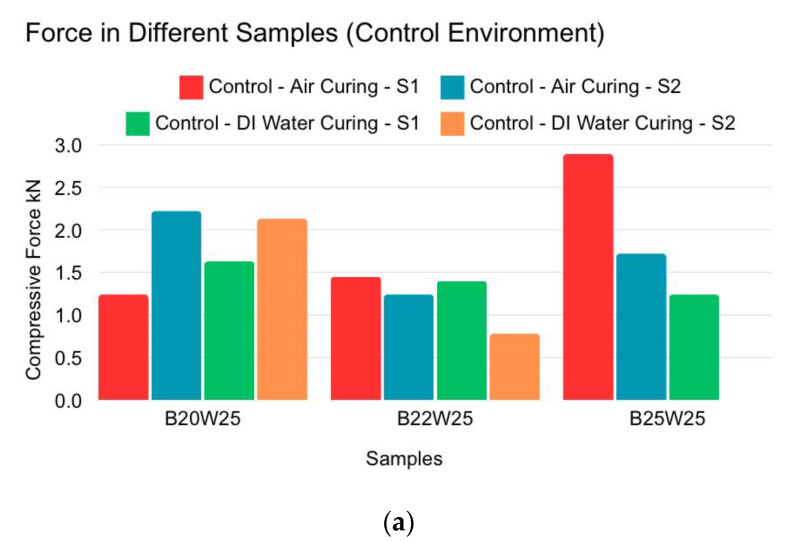

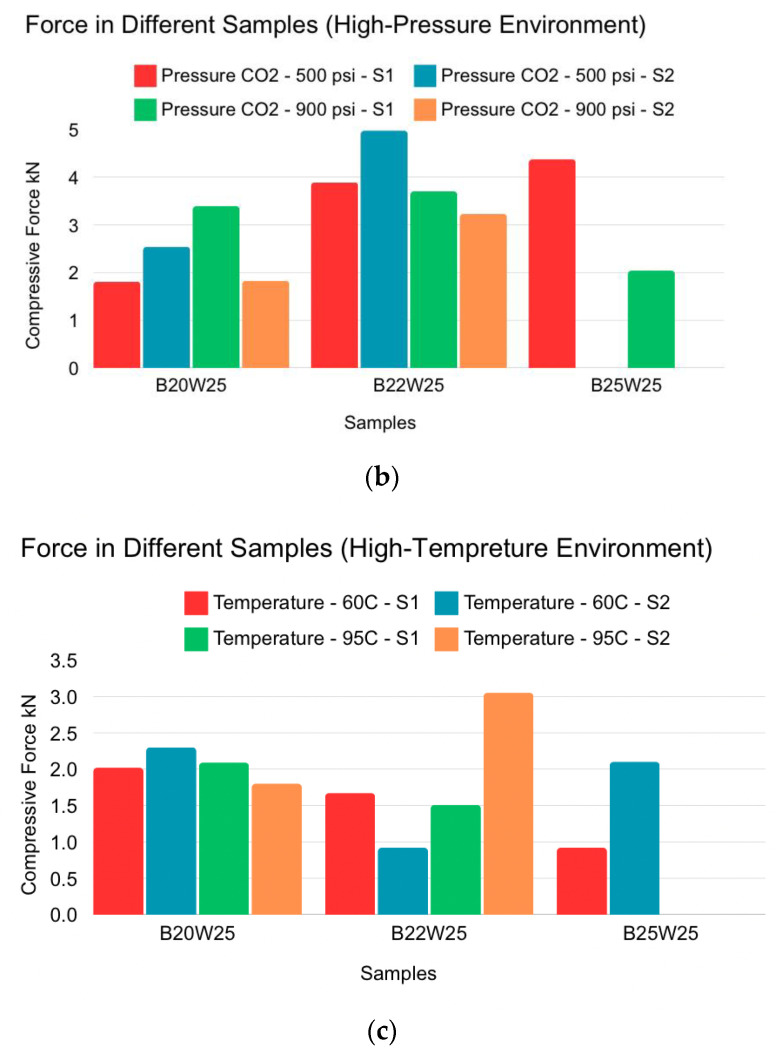
Spherical sample compression force for different samples. (**a**) Controlled environment (air curing and distilled water curing). (**b**) High-pressure environment (CO_2_ 500 psi and 900 psi). (**c**) High-temperature environment (60 °C and 95 °C).

Although one B25W25 sample provided the highest compressive force during air curing, it failed in DI water curing, indicating inconsistency. The B20W25 samples consistently provided the highest results on average, as shown in Figure 4a. The B22W25 samples exhibited higher compression force/strength as they became more compressed under pressure, becoming more concise, as shown in Figure 4b. The B25W25 samples failed in the compression test when pressurized to 900 psi. The B20W25 samples provided average results. In the high-temperature test, even though all the samples deteriorated, the B20W25 samples provided the highest force throughout, with results not differing much from the control samples, as indicated in Figure 4c.

**Figure 5:**
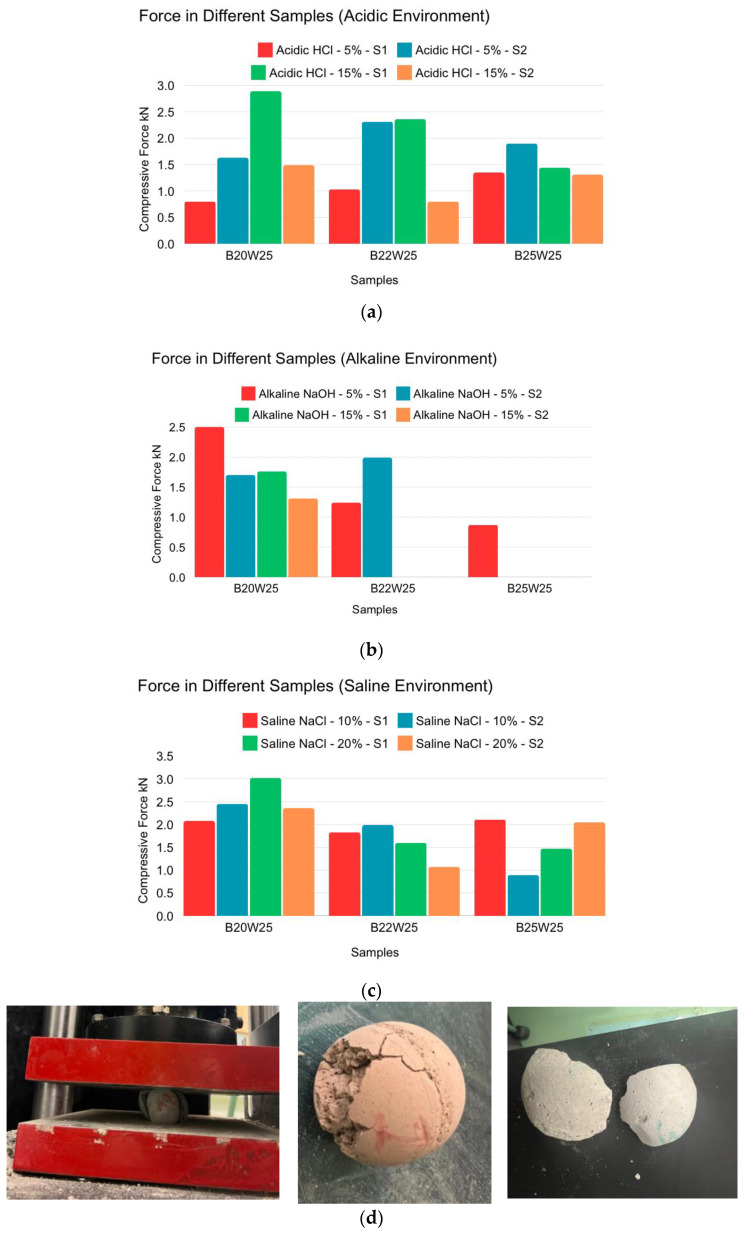
Spherical sample compression force in different samples. (**a**) Acidic environment (HCl 5% and HCl 15%). (**b**) Alkaline environment (NaOH 5% and NaOH 15%). (**c**) Saline environment (NaCl 10% and NaCl 20%). (**d**) Spherical sample failure shapes after compression testing.

The acidic environments, shown in Figure 5a, had a minor effect on the samples’ strength. On average, the samples had similar compression force values, with the B25W25 samples showing the lowest strength. For the B20W25 samples, the 5% concentration made them weaker than the 15% concentration. Alkalinity did not significantly change the strength of B20W25 samples, but it did affect the other mix design samples, as indicated in Figure 5b. Many samples did not survive the 15% concentration, but their performance was lower than the controlled compression force. The saline environment did not significantly affect the compression force of the B20W25 samples, but it did affect the other mix design samples, as showcased in Figure 5c.

**Figure 6:**
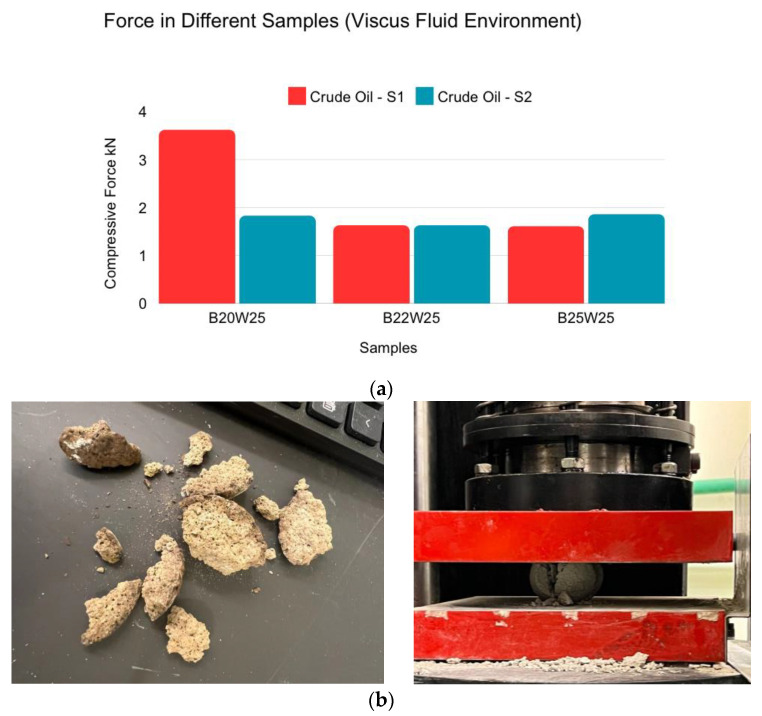
(**a**) Spherical sample compression force for different samples (viscus oil environment (crude oil)). (**b**) Final condition and failure shapes of spherical samples placed in viscus oil environment (crude oil) after compression test.

Crude oil had no effect on the strength of the samples overall, as indicated in Figure 6a. However, it made one B20W25 sample stronger as the oil made the outer layers of the sample more intact and compressed. To be precise, comparing the sample of the crude oil-exposed samples and air-cured samples in Figure 4a, the maximum value for B20W25 was 2.2 KN for sample S2 in air curing, and, in comparison, sample S1 in crude oil exposure yielded 3.7 KN. The other sample in air curing, sample S1, withheld 1.25 KN, whereas sample S2 in crude oil exposure withheld 1.9 KN. As shown in Figure 6b, after the outer layer of the sample’s crude oil was compressed and broken down, it was observed that the crude oil did not penetrate deep within the sample as the only darkened parts were the outer layers.

## 4. Discussion

### 4.1. Compression Test

Overall, all samples exhibited very low compression strength and failed under minimal load, with the highest strength recorded at approximately 1180 psi, far below the required minimum of 5000 psi [9]. Additionally, when looking at the spherical samples, with each group being in different environmental conditions, and comparing them to the control samples, consistent results were observed for B20W25 which was the more successful mix design. This goes hand in hand with the results found in the cubical samples’ strength results. On average, the alkaline test showed the most reduction in strength for all the samples, followed by an acidic environment and a high-temperature environment. B25W25 showed the least promising results, with many of the samples not succeeding in the environmental conditions. To compare the superior mix design B20W25 with the industry standard proppants, the highest compressive strength recorded was 1180 psi, while a typical sand proppant can withhold up to 5800 psi and a ceramic proppant from 7000 psi to 15,000 psi, as shown in Table 1 [9,15,16]. Thus, the fly ash proppant activated by an alkaline solution is not satisfactory for field utilization.

### 4.2. Future Directions

This research articulates a novel paradigm for proppant development, underscoring the potential advantages of integrating innovative materials and additives to augment mechanical properties. Future research could delve into a diverse array of fly ash types and binders, as well as alternative mixing methodologies and inventive approaches to binder incorporation. As fly ash is a byproduct, different batches will have different properties, as well as different types; thus, further investigation needs to be conducted linking the chemical composition of the fly ash with the proppant design and mechanical properties. When further understanding has been developed, enhancing the fly ash alkaline-activated proppant with additives can be looked at to reach the industry standard. Other binding and activating materials/solutions also need to be investigated besides the alkaline activators. Furthermore, an exploration of varied proppant geometries and utilization techniques could yield significant enhancements in performance across multiple applications. In sum, this study lays a robust foundation for ongoing inquiry and advancement within the realm of proppant technology.

## 5. Conclusions

This research explored the potential of fly ash as an alternative proppant material for hydraulic fracturing, mainly evaluating its compressive strength before and after testing the samples under different environmental conditions. However, the compression tests revealed that all mix designs had very low compressive strengths, far below the industrial requirement of 5000 psi, indicating that the current materials and mix designs are unsuitable for general industrial use [9]. However, the findings indicate that fly ash proppants have a potential in being a key ingredient in proppant design and development. The main limitation of this research is the lack of investigation of different types of fly ashes in order to understand if the main deficiency in the approach is the design of the proppant solely with fly ash or it is due to the fly ash used being insufficient. Additionally, more investigation needs to be conducted regarding the alkaline binders to know which activators are the most suitable with the fly ash used. Future research should emphasize the incorporation of innovative materials and additives, the experimentation with various types of fly ash and binders, and the exploration of alternative mixing methodologies and proppant geometries to enhance overall performance. The first actionable recommendation for future work is to test different types of fly ashes and binders as well as chemically analyzing the raw materials and synthesized products to understand further how a proppant compatible with industry standards can be made from fly ash. Although this study identified certain mechanical limitations, it provides a solid foundation for future investigations aimed at optimizing fly ash proppants. In summary, fly ash holds significant promise as a cost-effective and sustainable proppant material for hydraulic fracturing applications.

## Figures and Tables

**Table 1:** Conventional and novel fly ash proppant properties.

Proppant Type	Density (g/cm³)	Compressive Strength (psi)	Environmental Durability
Sand	2.65	Max 5800	Poor
Resin-Coated Sand	>2.65	6000–7000	Good
Ceramic	2.55–3.9	7000–15,000	Excellent
Novel Fly Ash	1.33–1.36	1180	Very Good

## Data Availability

The original contributions presented in this study are included in the article. Further inquiries can be directed to the corresponding author.

## Abbreviations

FA	Fly ash
B	Binder
W	Water
B20W25	Binder 20% weight ratio and water 25% weight ratio
B22W25	Binder 22% weight ratio and water 25% weight ratio
B25W25	Binder 25% weight ratio and water 25% weight ratio
SiO₂	Silicon dioxide
Al₂O₃	Alumina oxide
Fe₂O₃	Iron oxide
CaO	Calcium oxide
Na₂SiO₃	Sodium metasilicate
HCl	Hydrochloric acid
NaOH	Sodium hydroxide
NaCl	Sodium chloride
CO₂	Carbon dioxide

## References

[1] Chen T., Gao J., Zhao Y., Liang T., Hu G., Han X. (2022). Progress of Polymer Application in Coated Proppant and Ultra-Low Density Proppant. Polymers.

[2] Li H., Huang B. (2022). A new permeability model of fracture containing proppants. J. Nat. Gas Sci. Eng..

[3] Wu Z., Wu C., Zhou L. (2022). Experimental Study of Proppant Placement Characteristics in Curving Fractures. Energies.

[4] Dilireba T., Wang J. (2024). Effect of Proppant Damages on Fracture Conductivity and Long-Term Recovery in Shale Gas Reservoirs. Energy Fuels.

[5] Ding X., Wang T., Dong M., Chen N. (2022). Influence of Proppant Size on the Proppant Embedment Depth. ACS Omega.

[6] Li Q., Li Q., Han Y. (2024). A Numerical Investigation on Kick Control with the Displacement Kill Method during a Well Test in a Deep-Water Gas Reservoir: A Case Study. Processes.

[7] Li Q., Li Q., Wang F., Wu J., Wang Y. (2024). The Carrying Behavior of Water-Based Fracturing Fluid in Shale Reservoir Fractures and Molecular Dynamics of Sand-Carrying Mechanism. Processes.

[8] Guo B., Liu X., Tan X. (2017). Chapter 14—Hydraulic Fracturing. Petroleum Production Engineering.

[9] Zheng W., Silva S.C., Tannant D.D. (2018). Crushing characteristics of four different proppants and implications for fracture conductivity. J. Nat. Gas Sci. Eng..

[10] Xu F., Yao K., Li D., Xu D., Yang H. (2022). Study on the Effect of Acid Corrosion on Proppant Properties. Energies.

[11] Underdown D.R., Das K. (1985). New Proppant for Deep Hydraulic Fracturing. J. Pet. Technol..

[12] Wei X., Wang Y., Yang T., Song Y. (2023). A study on a new type of high-performance resin-coated sand for petroleum fracturing proppants. Coatings.

[13] Haydar R., Fakher S. Harsh Environmental Effects on Low Density Fly Ash Proppants. Proceedings of the Mediterranean Offshore Conference.

[14] Liao Z., Rabiee H., Ge L., Li X., Yang Z., Xue Q., Shen C., Wang H. (2024). Revealling pore microstructure impacts on the compressive strength of porous proppant based on finite and discrete element method. J. Mater. Sci. Technol..

[15] Pangilinan K.D., de Leon A.C.C., Advincula R.C. (2016). Polymers for proppants used in hydraulic fracturing. J. Pet. Sci. Eng..

[16] Kc B., Ghazanfari E., McLennan J., Frash L.P., Meng M. (2024). Evaluation of sintered bauxite proppant for binary enhanced geothermal systems. Géoméch. Geophys. Geo-Energy Geo-Resour..

[17] Rickards A.R., Brannon H.D., Wood W.D., Stephenson C.J. (2006). High Strength, Ultralightweight Proppant Lends New Dimensions to Hydraulic Fracturing Applications. SPE Prod. Oper..

[18] Helmy Y., Fakher S. (2024). Evaluating the Performance of Class F Fly Ash Compared to Class G Cement for Hydrocarbon Wells Cementing: An Experimental Investigation. Materials.

[19] Permatasari R., Sodri A., Gustina H.A. (2023). Utilization of Fly Ash Waste in the Cement Industry and its Environmental Impact: A Review. J. Penelit. Pendidik. IPA.

[20] Hemalatha T., Ramaswamy A. (2022). Coal-Based fly ash. Handbook of Fly Ash 2022.

[21] Sanjuán M., Argiz C. (2021). Fineness of Coal Fly Ash for Use in Cement and concrete. Fuels.

[22] Menéndez E., Argiz C., Recino H., Sanjuán M. (2022). Characterization of Mortars Made with Coal Ashes Identified as a Way Forward to Mitigate Climate Change. Crystals.

[23] Sanjuán M., Suarez-Navarro J.A., Argiz C., Estévez E. (2021). Radiation dose calculation of fine and coarse coal fly ash used for building purposes. J. Radioanal. Nucl. Chem..

[24] Menéndez E., Argiz C., Sanjuán M. (2021). Reactivity of Ground Coal Bottom Ash to Be Used in Portland Cement. J.

[25] Wu X., Huo Z., Ren Q., Li H., Lin F., Wei T. (2017). Preparation and characterization of ceramic proppants with low density and high strength using fly ash. J. Alloys Compd..

[26] Ding S., Jow J. (2022). Low-Density and High-Strength Fracking Proppant Made by High-Alumina Fly Ash. Coal Combust. Gasif. Prod..

[27] Zhang Z., Zhao M., Zhang Y., Liu C., Zhu W., Liu G., Yang Y., Sun G., Yang L. (2023). The solidification and volatilization behavior of heavy metal ions in ceramic proppant made from fly ash. Ceram. Int..

[28] Haydar R.R., Fakher S. Development of a Low Cost Environmentally Friendly Proppant with High Buoyancy for Hydraulic Fracturing Operations. Proceedings of the 57th U.S. Rock Mechanics/Geomechanics Symposium.

[29] Ren Q., Ren Y., Li H., Wu X., Bai W., Zheng J., Hai O. (2019). Preparation and characterization of high silicon ceramic proppants using low grade bauxite and fly ash. Mater. Chem. Phys..

[30] Ahmad S., Alam Ghazi M.S., Syed M., Al-Osta M.A. (2024). Utilization of fly ash with and without secondary additives for stabilizing expansive soils: A review. Results Eng..

[31] Nomani M., Shaquib O., Lone A.A. (2024). Environmental Implications of Fly Ash Management and Utilization: A Review of Laws, Policies, and Practices. Curr. World Environ. J..

[32] Das D., Rout P.K. (2023). A Review of Coal Fly Ash Utilization to Save the Environment. Water Air Soil Pollut..

[33] Suppiah R.R., Rahman S.H.A., Irawan S., Shafiq N. Development of New Formulation of Geopolymer Cement for Oil Well Cementing. Proceedings of the International Petroleum Technology Conference.

[34] Singh N.B. (2018). Fly Ash-Based Geopolymer Binder: A Future Construction Material. Minerals.

[35] Haruna S., Mohammed B.S., Wahab M.M.A., Kankia M.U., Amran M., Gora A.M. (2021). Long-Term Strength Development of Fly Ash-Based One-Part Alkali-Activated Binders. Materials.

[36] (2020). Standard Test Method for Compressive Strength of Hydraulic Cement Mortars (Using 2-in. or [50-mm] Cube Specimens).

